# The role of oligodendrocyte gap junctions in neuroinflammation

**DOI:** 10.1080/19336950.2019.1631107

**Published:** 2019-06-23

**Authors:** Christos Papaneophytou, Elena Georgiou, Kleopas A. Kleopa

**Affiliations:** a Neuroscience Laboratory, The Cyprus Institute of Neurology and Genetics and Cyprus School of Molecular Medicine, Nicosia, Cyprus; b Department of Life and Health Sciences, School of Sciences and Engineering, University of Nicosia, Nicosia, Cyprus; c Neurology Clinics, the Cyprus Institute of Neurology and Genetics, and the Cyprus School of Molecular Medicine, Nicosia, Cyprus

**Keywords:** Gap junction, oligodendrocytes, connexin, neuroinflammation, myelin

## Abstract

Gap junctions (GJs) provide channels for direct cell-to-cell connectivity serving the homeostasis in several organs of vertebrates including the central (CNS) and peripheral (PNS) nervous systems. GJs are composed of connexins (Cx), which show a highly distinct cellular and subcellular expression pattern. Oligodendrocytes, the myelinating cells of the CNS, are characterized by extensive GJ connectivity with each other as well as with astrocytes. The main oligodendrocyte connexins forming these GJ channels are Cx47 and Cx32. The importance of these channels has been highlighted by the discovery of human diseases caused by mutations in oligodendrocyte connexins, manifesting with leukodystrophy or transient encephalopathy. Experimental models have provided further evidence that oligodendrocyte GJs are essential for CNS myelination and homeostasis, while a strong inflammatory component has been recognized in the absence of oligodendrocyte connexins. Further studies revealed that connexins are also disrupted in multiple sclerosis (MS) brain, and in experimental models of induced inflammatory demyelination. Moreover, induced demyelination was more severe and associated with higher degree of CNS inflammation in models with oligodendrocyte GJ deficiency, suggesting that disrupted connexin expression in oligodendrocytes is not only a consequence but can also drive a pro-inflammatory environment in acquired demyelinating disorders such as MS. In this review, we summarize the current insights from human disorders as well as from genetic and acquired models of demyelination related to oligodendrocyte connexins, with the remaining challenges and perspectives.

## Introduction

Glial cells, including microglia, astrocytes, and oligodendrocytes, constitute a large fraction of the mammalian brain [1]. Oligodendrocytes are the myelinating cells of the central nervous system (CNS). Owing to their unique metabolic requirements and high degree of differentiation, oligodendrocytes are among the most susceptible cells of the CNS under various pathological conditions. They are the final product of a cell lineage that undergoes a complex and precisely timed program of proliferation, migration, differentiation, and myelination to finally produce the functionally important myelin sheath of axons that not only provides insulation to speed up conduction, but also essential metabolic and homeostatic support [2,3].

Oligodendrocytes are electrically and metabolically coupled through intercellular channels called gap junctions (GJs) with other oligodendrocytes (O/O junctions), as well as with astrocytes (O/A junctions). Furthermore, astrocytes are extensively coupled with each other (A/A junctions) creating a functionally interacting glial network over longer distances. This glial network of communication plays important roles in the homeostasis of brain function, exchange of metabolic substrates, coordination of brain activity, cell proliferation, migration, and differentiation [4,5]. In particular, oligodendrocyte GJs mediate the trafficking of ions and nutrients from the somas to the myelin layers and to the axons themselves and have proven to be essential for the development and survival of CNS myelin [6–8].

GJ channels are composed of connexins (Cx), a family of highly conserved integral membrane proteins that form hexamers around a central pore of hemichannels [9,10]. Each hemichannel on a cell membrane interacts in trans with another hemichannel on a closely apposed membrane that belongs to another cell to form the complete GJ channel allowing the transfer of molecules directly from one cell to another. Hemichannels can also be found unopposed, allowing the release of signaling molecules to the extracellular compartment, and may thus participate in glial transmission [11]. Different connexins are expressed by oligodendrocytes and astrocytes, i.e., oligodendrocytes primarily express Cx29, Cx32, and Cx47 [12,13], while astrocytes express Cx30 and Cx43 [14,15]. Amongst the glial Cxs, Cx43 and its oligodendrocytic coupling partner Cx47 are the most essential for maintenance of CNS myelin, especially in the white matter, as they form the majority of O/A GJs [16,17].

The importance of GJ-mediated connectivity in oligodendrocyte has been highlighted by human disorders associated with mutations in oligodendrocyte connexins. Mutations in the *GJB1* gene, that encodes Cx32, cause X-linked Charcot–Marie–Tooth disease (CMT1X), the second most common type of inherited demyelinating neuropathy, which is often associated with subclinical and sometimes clinical manifestations of CNS involvement as well [18]. Loss of function mutations in the *GJC2* gene encoding Cx47 result in hypomyelinating leukodystrophy Type 2 (HLD2) characterized by abnormal CNS myelin formation and progressive degeneration, as well as nystagmus, progressive spasticity, and ataxia [19–21]. Another disorder affecting the astrocytic Cx43, known as occulo-dental-digital dysplasia (ODDD) syndrome, presents among other systemic manifestations also with leukodystrophy [22], highlighting the importance of O/A GJ for the integrity of CNS myelin.

Motivated by the genetic disorders of oligodendrocyte connexins, and prominent inflammatory changes resulting from them, recent studies have also provided insight into their role in acquired demyelinating CNS disorders, in particular, multiple sclerosis (MS) and related experimental models [23–26]. These studies revealed that oligodendrocyte connexins are not only disrupted in MS lesions and beyond, but they also appear to have a regulatory role in neuroinflammation as their absence further exacerbates inflammatory demyelination [27]. In this review, we highlight the important role of oligodendrocyte connexins, in particular, Cx32 and Cx47 in the normal function of CNS, by introducing insights from genetic and experimental demyelination models. Moreover, we discuss the roles of these Cxs in both inherited and acquired demyelinating human diseases in the context of neuroinflammation.

## The expression pattern of connexins in oligodendrocytes

Oligodendrocytes express three Cxs, i.e. Cx29, Cx32, and Cx47 [12] and form GJs mostly with astrocytes (O/A) [14,28], as well as with other oligodendrocytes (O/O) [29,30]. O/O coupling is facilitated by GJs in homotypic configurations with homomeric hemichannels containing Cx32 or Cx47 [17]. Astrocytes express different connexins, Cx30 and Cx43, and thus O/A channels are heterotypic, composed of either Cx43–Cx47 or Cx30–Cx32 channels [14,17,31,32]. Due to compatibility features, neither Cx32 couples with Cx43 in astrocytes nor Cx47 with Cx30. Beyond oligodendrocytes, astrocytes are extensively connected with other astrocytes either with Cx43-Cx43 or with Cx30-Cx30 channels, the latter being more prominent in the gray matter [25,31].

Cx32 is expressed mainly by white matter oligodendrocytes and is localized in the myelin sheath of large diameter fibers forming most intracellular or “reflexive” GJs within the myelin sheath [33]. In certain white matter areas composed mostly of thinly myelinated small diameter fibers, such as the corpus callosum and the optic nerve, Cx32 is absent [12]. In gray matter, Cx32 is expressed mainly in perikarya and proximal processes of oligodendrocytes [12,13,34] and forms intercellular O/O homotypic GJs or heterotypic O/A GJs with astrocytic partner Cx30. Cx32 is also the major GJ protein in peripheral myelinating cells, the Schwann cells, forming intracellular GJs in non-compact myelin areas including the paranodal myelin loops and Schmidt-Lantermann incisures [33].

Cx47 is expressed at earlier developmental stages preceding CNS myelination [35] compared to Cx32 expression that coincides with myelination [33]. It is found in all oligodendrocytes throughout the CNS in both white and gray matter. It is mainly localized in the perikarya and proximal processes of the myelinating cells, as well as in oligodendrocyte precursors cells (OPCs) [24] and forms the majority of intercellular GJs with other oligodendrocytes (O/O) and with astrocytes (A/O) [12–14,36,37]. Cx32 and Cx47 may be colocalized in some GJ plaques on oligodendrocyte somata but they do not appear to participate in heteromeric channels.

Finally, Cx29 in rodent oligodendrocytes [34], and its human ortholog Cx31.1 [38], appear to form only hemichannels in the innermost myelin layer of small myelinated fibers at juxtaparanodal localization, in close proximity to the axonal potassium channels Kv1.1 and Kv1.2. Cx29 is expressed in the gray matter and in certain white matter areas including the corticospinal tract, optic nerve, and corpus callosum, where Cx32 is typically absent [12]. Thus, Cx29 and Cx32 appear to be expressed in different white matter areas in a mutually exclusive pattern, dictated by the diameter of myelinated fibers. As it forms only hemichannels, Cx29 rarely colocalizes with any of the other glial connexins [14].

Cx29 hemichannels, as well as Cx32 and Cx47 GJ channels in oligodendrocytes, are essential for spatial buffering of K^+^ in response to neuronal activity; failure of this function leads to myelin swelling and subsequent axonal degeneration [39]. In addition, oligodendrocytic GJs facilitate the transportation of nutrients and ions from oligodendrocyte cell body to myelin layers [2].

## Genetic disorders associated with oligodendrocyte connexins

### Hypomyelinating leukodystrophy type 2 (HLD2)

*GJC2*/Cx47 mutations cause HLD2, also known as Pelizaeus-Merzbacher-like disease (PMLD), an early onset dysmyelinating disorder characterized by nystagmus, psychomotor retardation, progressive spasticity and cerebellar signs [19]. At least one mutation has been associated with a milder phenotype of hereditary spastic paraplegia, likely because it retains some of the Cx47 function [36]. Different homozygous and compound heterozygous *GJC2* mutations affecting Cx47 were identified in consanguineous and non-consanguineous families with autosomal recessive inheritance [19,20]. Homozygous deletions leading to frameshift in the *GJC2* gene were also reported to cause a similar phenotype [40,41], suggesting a loss-of-function effect. Furthermore, homozygous mutations within the *GJC2* promoter region affecting the key transcription factor SOX10 binding site have been identified in HLD2 patients [42,43]. These results suggest that the SOX10-to-*GJC2* transcriptional dysregulation is a cause of HLD2, as well as that *GJC2,* may be in part responsible for the central hypomyelination caused by *SOX10* mutations.

Compared with “classical” Pelizaeus–Merzbacher Disease (PMD) with *PLP1* mutations, HLD2/PMLD patients with *GJC2* mutations initially have a milder phenotype, with higher achieved cognitive levels and speech capacity. However, neurologic deterioration starts earlier and progresses faster, with shorter interval to loss of speech capacity, as well as loss of ambulation and wheelchair dependency [44]. One hypothesis to account for this clinical difference is that loss of Cx47-containing GJs leads to more rapid axonal degeneration than does the loss of proteolipid protein (PLP) [45]. Axonal degeneration likely correlates with the severity of the phenotype in HLD2/PMLD, similar to CMT1X peripheral neuropathy, as well as other CNS white matter disorders including PMD and MS [46–48].

### X-linked Charcot-Marie-Tooth disease

CMT1X is the second most common form of hereditary motor and sensory neuropathy and is caused by more than 400 different mutations in the *GJB1* gene that encodes Cx32 [48,49]. Many Cx32 mutants fail to form functional GJs, or form GJs with abnormal biophysical properties [49]. CMT1X peripheral neuropathy shows a typical onset in late childhood and adolescence and affects men earlier and more severely, while heterozygous women may remain asymptomatic or develop milder manifestations later in life [18].

In addition to peripheral neuropathy, several *GJB1* mutations have been associated with subclinical-electrophysiological, or even clinical and/or magnetic resonance imaging (MRI) findings of chronic or transient CNS involvement [50–53]. Moreover, patients with R22Q, T55I, R75W, E102del, V139M, R142W, R164W, R164Q, C168Y, or V177A mutations have developed the striking picture of an acute, transient encephalopathy associated with MRI changes in CNS myelin, often “triggered” by travel to high altitudes, intense physical activity, or acute infections [54–59]. These electrophysiological findings have not been found in patients with deletion of the entire *GJB1* open reading frame [60] and therefore it has been proposed that these mutants may cause a gain of abnormal function. However, when the mutants T55I and R75W were transgenically expressed in oligodendrocytes, they had no apparent trans-dominant effect on co-expressed Cx47 [61]. Furthermore, at least one case with a start codon mutation in *GJB1* and documented complete loss of Cx32 expression was also associated with transient CNS manifestations, suggesting that gain-of-function effects are not necessary for the CNS manifestations of CMT1X [62]. Further studies to elucidate the consequences of *GJB1* mutations, in particular in the CNS, were subsequently undertaken and discussed below [63,64].

## Genetic models of oligodendrocyte connexins and gene replacement studies

In order to better understand the role of oligodendrocyte connexins in CNS myelination, various genetic models lacking one or more oligodendrocyte or astrocyte connexins have been generated [4,65]. Mice lacking Cx47 developed only mild CNS pathology, probably due to overlapping function with Cx32 [7]. Moreover, Cx47 knockout (KO) mice are fertile and do not show anatomical or behavioral abnormalities [6]. However, under stress conditions, or with additional deletion of the Kir1.4 channel localized at astrocyte end feet and participating in the same homeostatic pathway as oligodendrocyte-astrocyte GJs, beginning pathology and vacuolation was observed along the optic nerve [39]. Likewise, although mice lacking Cx32 develop a progressive peripheral neuropathy starting after 3 months of age [66,67], they present only very mild CNS myelination defects consisting of reduced myelin density in certain white matter areas such as the dorsal and ventral funiculus of the spinal cord [61,68].

However, deletion of both major oligodendrocyte connexins in Cx32/Cx47 double-knockout (dKO) mice leads to abnormal movements and seizures associated with vacuolated myelin and axonal degeneration in the CNS [6,7]. These dKO mice with complete loss of oligodendrocyte GJ connectivity develop a progressive, coarse action tremor during the third postnatal week followed by tonic seizures, which increase in frequency and severity until the animals die, usually during the sixth postnatal week. Pathologically, at one month of age, they show severe CNS demyelination, axonal degeneration, and apoptosis of oligodendrocytes in the spinal cord funiculi and in the optic nerves, confirming that GJ connectivity is vital for oligodendrocytes and the myelin they form [6,7].

Of particular interest is that mouse models with deficient oligodendrocyte GJ connectivity present marked inflammatory changes. In particular, the Cx32/Cx47 dKO model was found to develop increased B- and T-cell infiltrates with upregulation of pro-inflammatory pathways, astrogliosis, microglia activation, and macrophage infiltrates [69–71]. Similar inflammatory changes have been already a well-recognized feature of peripheral nerve pathology in Cx32 KO mice [72,73] and indicate that the loss of connexins in myelinating cells has a pro-inflammatory effect both in the CNS and PNS (discussed further below).

Pathological changes in Cx32 and Cx47 KO mice and related human disorders result from cell-autonomous mechanisms. This has been further demonstrated by transgenic replacement experiments and with cell-targeted gene therapy approaches. Transgenic replacement of Cx32 expression in Schwann cells of Cx32 KO mice driven by the Schwann cell-specific myelin protein zero (MPZ) promoter resulted in full rescue of the peripheral neuropathy, confirming the cell-autonomous mechanisms in CMT1X neuropathy [74]. Likewise, transgenic expression of human Cx32 in oligodendrocytes of Cx32/Cx47dKO mice driven by the PLP promoter resulted in almost complete rescue of behavioral abnormalities at 1 month of age and prevented the severe CNS demyelination and early mortality. Oligodendrocyte GJ connectivity was re-established through homotypic O/O Cx32-Cx32 and heterotypic O/A Cx32-Cx30 channels [69]. In the same study, PLP promoter-driven expression of Cx32 in Schwann cells also rescued the peripheral neuropathy in Cx32 KO mice.

Utilizing the Cx32/Cx47 dKO model of HLD2, we examined whether *GJC2/Cx47* gene replacement specifically in oligodendrocytes would provide a therapeutic approach for this type of leukodystrophy. To develop an oligodendrocyte-targeted gene delivery we used a chimeric adeno-associated viral (AAV1/2) vector previously shown to efficiently infect oligodendrocytes [75] carrying the human *GJC2/Cx47* gene under the control of the 1.9 kb oligodendrocyte-specific myelin basic protein (MBP) promoter. The AAV.MBP.EGFP vector was injected into the internal capsule area of the left hemisphere in postnatal day 10 (P10) mice. At P30 we detected a maximum of EGFP positive cells in the corpus callosum and the internal capsule, with lower expression rates also in the striatum, hippocampus, midbrain, superficial layers of the pons, olfactory bulb, and basal forebrain. Viral expression extended also to the contralateral right hemisphere including the corpus callosum and in extensive white matter areas of the cervical and thoracic spinal cord. The intracranial EGFP expression showed a rostro-caudal spread of 7.61–0.68 mm and maximum lateral spread 4.61–0.66 mm with total expression volume of 46.00–7.93 mm^3^ [70].

Similar to the expression of the mock vector, delivery of the AAV.MBP.Cx47myc therapeutic vector in the internal capsule area of P10 Cx47 KO mice resulted in widespread expression of Cx47 in a high percentage of oligodendrocytes in the internal capsule and corpus callosum as well in the striatum, hippocampus, olfactory bulb, midbrain, pons and cervicothoracic spinal cord with expression ratios ranging from 32% to 43% on average. Virally expressed Cx47 was restricted to oligodendrocytes and showed the characteristic formation of GJ-like plaques in the cell bodies and proximal processes [70]. Using this approach, a treatment trial in dKO mice resulted in a significant therapeutic benefit compared to mock-treated or untreated mice at one month of age by re-establishing oligodendrocyte GJ connectivity as demonstrated by functional dye transfer analysis. There was a significant improvement in motor performance and coordination. Survival was modestly prolonged to 58 ± 4.7 days compared to 41 ± 3.6 days in mock-treated mice, with clearly no complete phenotype rescue, likely due to the severity of this model. However, immunofluorescence and morphological analysis at P30 revealed both in brain and spinal cord significant improvements in myelination and oligodendrocyte apoptosis, as well as in astrogliosis, microglia activation, and macrophage infiltrates, which are characteristic of this HLD2 model [70].

A Schwann cell-specific gene therapy approach has also been used to rescue the peripheral neuropathy in Cx32 KO mice. Driven by the same MPZ promoter shown to transgenically rescue this phenotype [74], the human *GJB1* gene was delivered by direct intraneural injection into sciatic nerves and led to re-establishment of Cx32 expression in paranodal myelin areas and improvement of the pathological changes in the injected nerve [62]. Subsequently, lumbar intrathecal delivery of human Cx32 resulted in a more widespread expression of Cx32 in the PNS and led to functional and pathological improvement in the Cx32 KO mouse model of CMT1X [76]. This approach was also applied in transgenic models expressing different Cx32 mutations associated with CMT1X on a Cx32 KO background, including the ER-retained T55I and the Golgi-retained R75W and N175D Cx32 mutations. This study showed that the therapeutic benefit previously found in Cx32 KO mice could be reproduced in T55I KO but not in N175D KO or R75W KO mice [77]. Virally delivered wild-type (WT) Cx32 could be detected in the non-compact myelin of T55I KO mice, but only rarely in N175D KO or R75W KO mice, suggesting dominant-negative effects of the R75W and N175D mutants but not of the T55I mutant on co-expressed WT Cx32 [77]. Further, *in vitro* Cx32 WT-mutant co-expression studies demonstrated directly that certain Golgi-retained CMT1X mutants may interfere with expression and intracellular trafficking of WT Cx32 [78], and could thus potentially interfere with gene addition therapy for CMT1X.

## Connexin pathology in models of neuroinflammation

The strong inflammatory reaction in the CNS of connexin deficient mice (above) suggests that oligodendrocyte GJs may play a role in acquired neuroinflammatory disorders [69–71]. In Cx32 KO mice extensive studies of peripheral nerves demonstrated progressive inflammatory changes that may contribute to disease progression [72,73]. We have also observed increased microglia activation in the CNS of Cx32 KO mice at baseline [23].

### Modeling inflammation-induced CNS manifestations of CMT1X

In order to understand the cellular mechanisms underlying the CNS phenotypes in patients with CMT1X (above), we used a model of systemic inflammation induced by lipopolysaccharide (LSP) injection. We compared the effects of LPS-induced systemic inflammation in Cx32 KO mice expressing the T55I mutant (KO T55I) with WT and Cx32 KO mice [63]. We assumed that dominant effects of the T55I mutant might only become obvious under stress conditions that mimic the metabolic and inflammatory events leading to encephalopathy in CMT1X patients [55–58]. The LPS model was characterized by early sickness behavior and transient weight loss, and showed that Cx32 KO mice are more vulnerable to neuroinflammation than WT mice, while KO T55I mice show even more vulnerability with exacerbated motor dysfunction compared to simple Cx32 KO or WT mice, suggesting additional gain-of-function mechanisms in the presence of the T551 mutant [63]. A combination of cellular alterations, both mutant-related but also independent from the mutant, likely contributed to disrupt oligodendrocyte integrity. Downregulation of astrocytic Cx43 as a non-specific response to inflammation leading to reduction of Cx47-Cx43 O/A GJ channels appeared to cause impaired oligodendrocyte connectivity to the glial GJ network that was more detrimental in the absence of Cx32 in the KO mice. Moreover, in the presence of the ER-retained T55I mutant in the T55I KO mouse, there was additional evidence of ER-stress response that could exacerbate further CNS dysfunction.

Additional studies to understand why certain CMT1X mutations are associated with CNS manifestations while others are not, showed that “PNS-only” mutants retained some ability to form functional GJs, while several “CNS” mutants either formed no morphological GJ plaques when expressed *in vitro*, or if they did, they produced little or no detectable junctional coupling [64].

### Oligodendrocyte connexin pathology in experimental autoimmune encephalomyelitis

Further studies of connexin pathology were focused on the experimental autoimmune encephalomyelitis (EAE) model, the most commonly used experimental model of MS, in order to gain insights into the time‐course and molecular mechanisms of inflammatory demyelination [79]. In both EAE and MS, demyelinating lesions develop predominantly in the CNS white matter associated with infiltrating T cells, macrophages, and B cells. In addition, foam cell-like macrophages containing phagocytosed hydrophobic myelin debris have been demonstrated within active lesions. In the EAE model ascending hind limb paralysis is associated with inflammation and demyelination of axonal tracks mainly in the lumbar-sacral spinal cord. Oligoclonal IgG can be found in the CSF of EAE mice as in MS patients [80].

Cx47 and Cx32, the only known connexins that form full GJ channels in oligodendrocytes [6,7,12] were found to be reduced in recombinant myelin oligodendrocyte glycoprotein (rMOG) induced EAE in WT mice [23]. Connexins were lost early on during acute EAE stages within but also around demyelinated areas, and even away from lesions. Their reduction persisted at later stages of EAE, suggesting a lasting disruption of oligodendrocyte connectivity. In contrast, Cx43, the main astrocytic connexin forming most O/A GJs, at least in the white matter [31,81], was disrupted in acute EAE stages but increased above normal in chronic EAE, reflecting astrogliosis [23]. As with the LPS model, Cx32 KO mice showed a more severe clinical phenotype and more inflammation as well as more extensive demyelination compared to WT mice when subjected to EAE induction [23].

The mechanism of connexin alterations in oligodendrocytes remains unclear, but several insights were obtained from the study of different stages of the rMOG EAE model, and subsequently of other models as well. Analysis of Cx43 expression, the major astrocytic partner of Cx47, at 14 and 28 dpi, showed a marked loss of Cx43 GJ plaques within and around demyelinating lesions and in a gradient fashion in the surrounding WM while GFAP immunoreactive astrocytic processes were intact but devoid of Cx43. The loss of Cx47 GJs in acute EAE stages coincided with the loss of Cx43 GJs in astrocytes, while at later stages increased expression of Cx43 was associated with only partial recovery of Cx47 GJs. Supporting the immunostaining results, immunoblot analysis showed that Cx47 protein levels were not reduced in acute EAE, suggesting a redistribution of Cx47 away from the membrane and diffusion intracellularly. In contrast, Cx43 levels were significantly reduced at 14 and 28 dpi.

In chronic EAE stages at 50 dpi, Cx43 immunoreactivity increased above normal levels within lesions and colocalized with dense GFAP immunoreactivity reflecting astrogliosis [23]. By immunoblot Cx43 levels also increased above naïve levels at 50 dpi, while Cx47 levels eventually dropped and remained reduced despite increasing levels of Cx43. Analysis of mRNA levels confirmed the downregulation of Cx43 in early EAE, while oligodendrocyte connexin genes were minimally or not at all downregulated, again indicating that the disruption of A/O GJs was rather secondary to changes occurring in astrocytes [23]. Similar upregulation of Cx43 in chronic astrogliosis has also been described in CNS injury models [82–86], while loss of Cx43 GJs in acute pathology has been reported in other EAE models [87–89]. It is likely mediated by proinflammatory cytokines [90,91], and may be a manifestation of a non-specific activated astrocyte phenotype [89,92–94], as it was also observed in our CFA controls [23].

Thus, neither increased degradation, nor reduced expression of Cx47 occurs in acute EAE, but rather redistribution from GJ plaques to the intracellular compartment, which may be secondary to the loss of Cx43, the main partner of Cx47 in heterotypic O/A GJs (above). Similar reduction of Cx43 protein and RNA levels has been shown in the acute phase of MHV-A59-induced viral infection model leading to a persistent downregulation of Cx47, associated with demyelination [95]. Furthermore, double deficiency of Cx43 and Cx32 in mice induces loss of Cx47-mediated channels, whereas Cx47 mRNA levels remain unaltered [32]. A mechanistic explanation for these observations has been provided by May et al. [96], who showed that the presence of Cx43 at the astrocyte cell membrane is necessary and sufficient for normal expression, phosphorylation, and stability of Cx47 on the opposing oligodendrocyte cell membrane, regardless of whether Cx43 forms functional GJs. Loss of astrocytic Cx43 in acute inflammatory models is therefore likely to result in diffusion of Cx47 away from oligodendrocyte cell membrane and disruption of GJ communication. Finally, Cx43 plays multiple metabolic and signaling roles in astrocytes that can affect oligodendrocytes independently of GJ channels [97]. Deletion of Cx43 in astrocytes decreases extracellular glucose levels and inhibits OPC proliferation [11]. Cx43 can bind to many proteins involved in downstream cell signaling pathways, including various kinases and phosphatases [98], affecting inflammatory processes.

**Figure 1.**
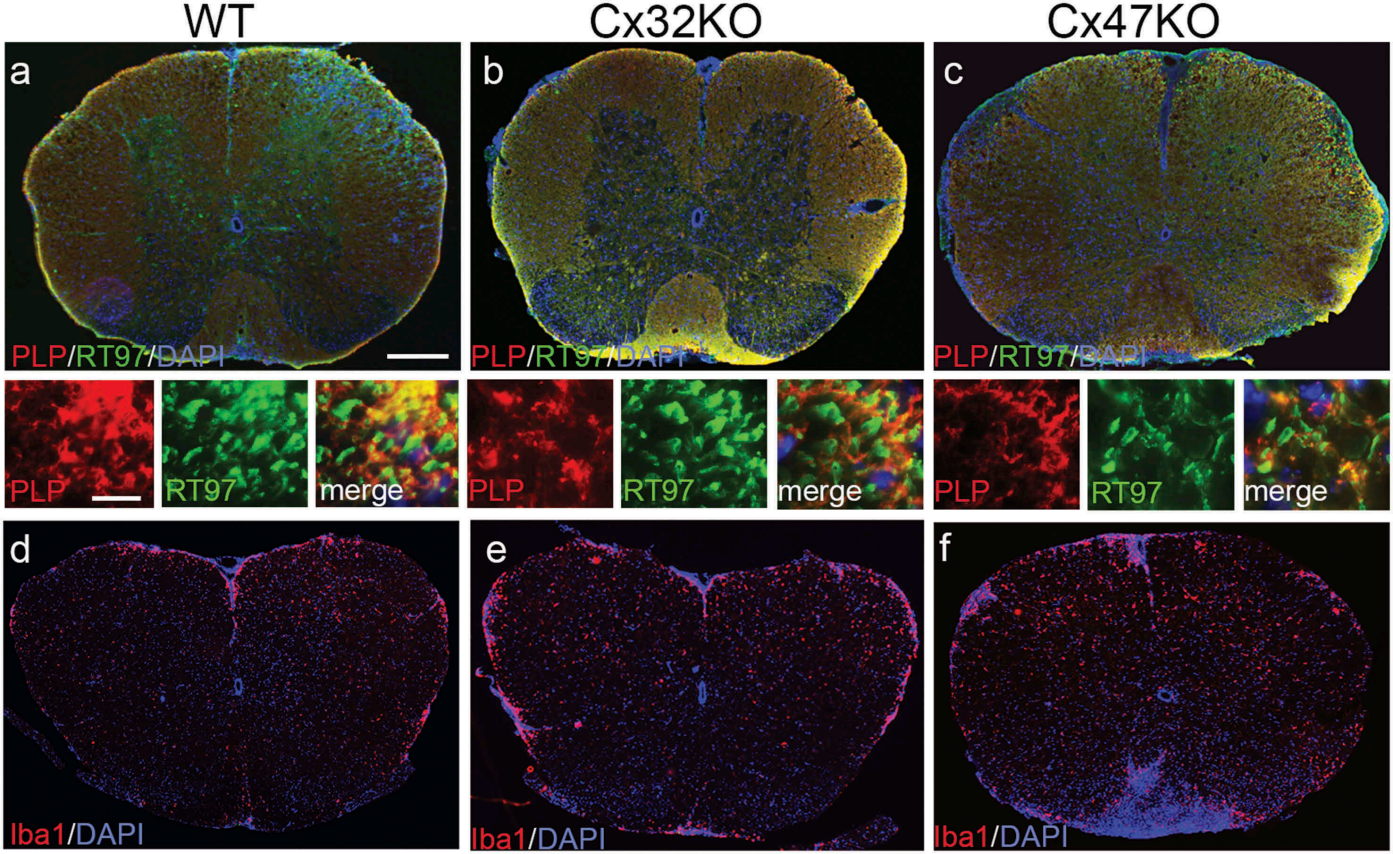
Pathological features of EAE in connexin mutants. These are representative images of whole lumbar spinal cord sections from EAE mice at 24 days post immunization (dpi), double stained as indicated with antibodies to myelin marker proteolipid protein (PLP) (red) to quantify demyelination, and axonal neurofilament marker RT97 (green) to quantify axonal loss (merged overview images and higher magnification images with separate channels underneath) (**a-c**), or for microglial marker Iba-1 (red) (**d-f**). Cell nuclei are stained with DAPI (blue). Areas of demyelination as well as diffuse microglia activation appear more extensive in connexin deficient mice, especially in the Cx47 KO. Scale bars in a= 100 μm; in insets = 10 μm.

To further examine whether the loss of either Cx47 or Cx32 in oligodendrocytes could affect the outcome of inflammation and myelin loss, and is not merely a side effect of this process, we induced EAE using the MOG_35-55_ peptide in fully backcrossed Cx32 KO and Cx47 KO mice and compared their outcome with WT (C57BI/6 N) mice. These studies revealed that mice lacking either Cx32 or Cx47 were more susceptible to EAE compared to WT mice. Specifically, Cx47 KO mice developed EAE more rapidly while the EAE phenotype was more severe in Cx47 KO mice compared with Cx32 KO mice and in turn, both connexin mutants showed more severe manifestations compared with WT mice. Moreover, pathological analysis revealed that Cx47 KO mice developed more myelin and axonal loss than did WT and Cx32KO mice [27]. Extensive microglia/macrophage activation with increased Iba-1 immunoreactivity was observed in all EAE groups, but this inflammatory activity was significantly greater in Cx47 KO EAE mice (Figure 1).

Further experiments confirmed that several parameters of EAE pathogenesis, including the breakdown of the blood-spinal cord barrier (BSCB), the infiltration of spinal cord tissue by inflammatory cells, and oligodendrocyte apoptosis were more severe in Cx47 KO mice than in Cx32 KO and WT mice, while differences between Cx32 KO and WT were generally smaller. Expression profiling analysis revealed that several pro-inflammatory cytokines were higher at the peak of inflammation in the Cx47 KO mice and persisted at later stages of EAE in contrast to reduction of their levels in WT EAE mice. These results confirm that oligodendrocyte GJs may play a regulatory role in inflammatory conditions and that their loss has a proinflammatory effect, a mechanism that may be directly relevant for MS pathogenesis and progression [27].

Several reasons may explain why loss of Cx47 increases oligodendrocyte susceptibility to CNS inflammation much more that the loss of Cx32 [27]. As outlined above, Cx47 is normally expressed in all oligodendrocytes in the CNS [6,7,12] while Cx32 is expressed only in a subset of oligodendrocytes myelinating larger diameter fibers [12,34]. Thus, a smaller population of oligodendrocytes might be affected by loss of Cx32 than by loss of Cx47. Furthermore, Cx47 forms the majority of the functionally essential O/A GJs throughout the CNS. Finally, the expression of Cx47 appears to be an earlier event in oligodendrocyte cell lineage differentiation, appearing as early as at postnatal day 7, and peaking around day 14–25 [7,35], preceding the expression peak of other myelin-related proteins including Cx32 [33]. Thus, Cx47 likely plays a more important role in OPC differentiation. The more important functional role of Cx47 in the CNS compared with that of Cx32 is also highlighted by the fact that patients with mutations in Cx47 develop the severe hypomyelinating leukodystrophy HLD2 [19], while patients with Cx32 mutations develop only subclinical or transient encephalopathy under conditions of additional metabolic stress (above) [58,62].

One of the main features of exacerbated CNS pathology following EAE induction, especially in Cx47 deficient mice, was the increased infiltration by inflammatory leukocytes associated with disruption of blood–brain barrier (BBB)/BSCB. Disruption of the BBB/BSCB is the histopathological hallmark of MS and EAE [99] and also a clinically relevant factor as it represents the initial stage of MS lesion formation [100,101]. Both BBB and BSCB are responsible for the correct functioning and homeostasis of the CNS by regulating cellular and molecular trafficking between blood and brain/spinal cord parenchyma [102]. OPCs have been implicated in the regulation of BBB/BSCB through the release of soluble factors such as TGF-β [103,104]. Since OPCs express both Cx47 [24] and Cx32 [105], the disruption of their GJ network integration in mutant mice may alter their regulatory effects on BBB/BSCB function. Furthermore, disruption of the majority of O/A GJs in Cx47 KO mice may indirectly dysregulate astrocyte responses to inflammatory signals.

Whether a direct involvement of Cx47 in the peripheral cellular immune response could contribute to the exacerbated EAE pathology in the CNS of Cx47 KO mouse remains to be clarified. Cx47 expression in the lymphatic epithelium [106,107] suggests a possible role in peripheral immune reactions. This could be one of the reasons for the heightened B-cell response in Cx47 KO EAE brains, since the pathway from brain lymphatics to draining cervical lymph nodes may modulate the CNS immune response [108]. Studies in MS patients revealed that clonal autoreactive B-cells mature in the cervical lymph nodes which collect from the brain lymphatics and can transmigrate freely into the CNS across the tissue barrier [109]. However, the possibility of non-oligodendrocyte expression of Cx47 as a contributor to increased neuroinflammation remains to be explored in future studies, for example, by generating and studying conditional Cx47 KO mice with oligodendrocyte-specific loss of Cx47.

## Glia connexin pathology in multiple sclerosis

**Figure 2.**
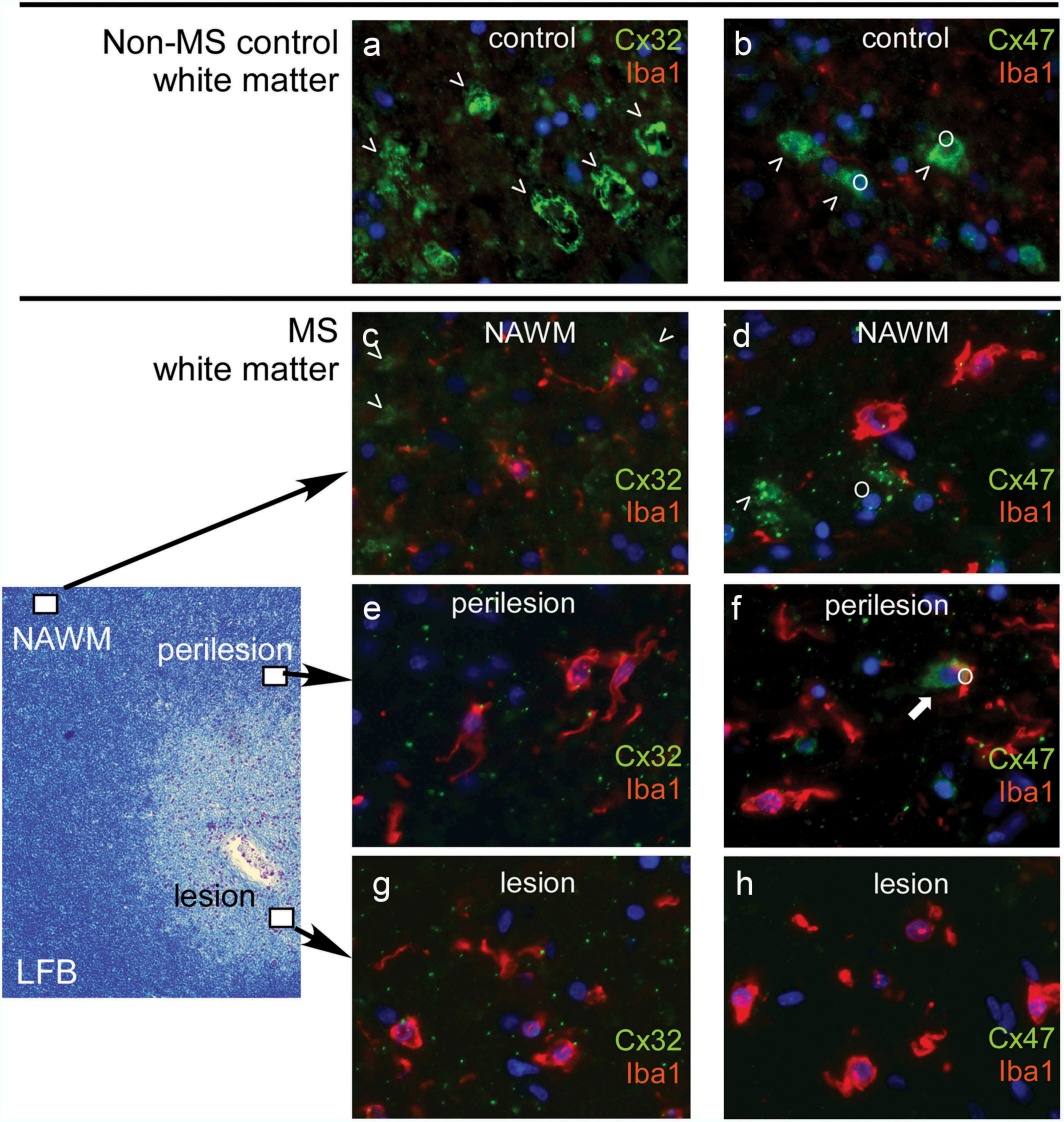
Expression of oligodendrocyte connexins in multiple sclerosis (MS) brain. Images of the white matter of a non-multiple sclerosis control (**a**- **b**) compared to a multiple sclerosis patient (**c**- **h**) are shown. Low magnification of MS brain white matter stained with Luxol fast blue (LFB) is shown on the left to indicate the location of the high magnification immunofluorescence pictures shown on the right. Antibodies to oligodendrocyte connexins as indicated (green) were combined with the Iba1 antibody labeling microglia (red). Cell nuclei are stained blue. The normal expression of Cx32 along large myelinated fibers in non-MS control brain (**a**) is significantly reduced, not only within and around lesions but also in NAWM in MS brain (**c, e**, **g**), associated with prominent microglia activation. Likewise, Cx47 expression mainly in cell bodies and proximal processes of oligodendrocytes (o) shown in non-MS brain (**b**) is reduced in and around MS lesions whereas, in contrast to Cx32, it appears preserved in NAWM (**d, f**, **h**).

The emerging roles of glia connexins have generated interest into their possible involvement in common acquired demyelinating disorders, especially MS, that is characterized by focal areas of inflammation in the CNS leading to demyelination, axonal loss and reactive astrocytosis [79]. MS is also associated with pathological changes in normal-appearing white matter (NAWM). Analysis of post-mortem human brain has demonstrated extensive alterations of glial GJ formation in MS, both within WM lesions as well as in the NAWM [24,26] and cortex [25]. To examine the role of oligodendrocyte Cx in MS pathology, we analyzed the Cx expression in post-mortem samples from MS patients and non‐MS controls after characterization of inflammatory load and demyelination, as well as determination of NAWM and lesion stages. Quantification of the inflammatory load assessed by Iba1 immunoreactivity confirmed higher levels of inflammation in MS compared with non-MS control brain tissue. Moreover, we found significant alterations in glial connexins not only in lesions but also extending into the NAWM [24] (Figure 2).

Compared with non-MS controls, the expression of Cx32 was reduced within and around MS lesions. Furthermore, in the NAWM, Cx32 GJs were significantly reduced along myelinated fibers. The disruption of Cx32 immunoreactivity along myelinated fibers, including the typical paranodal localization, occurred especially in areas with intense inflammation and was specific, while other myelin proteins were not reduced, and PLP immunoreactivity was preserved. Counting of Cx32‐immunoreactive GJ plaques confirmed a significant reduction in MS NAWM compared with controls, and a gradient of further reduction towards lesions.

In contrast to Cx32, Cx47 showed increased expression mainly in OPCs with a modest increase in Cx47 protein levels in MS NAWM. However, OPCs showed only limited connectivity to astrocytes. Astrocytes appeared reactive especially in chronic lesions, upregulated Cx43 expression, but formed mostly Cx43-Cx43 GJs with other astrocytes and reduced Cx43-Cx47 GJs with oligodendrocytes. Similar to Cx32, Cx47 GJ plaques showed a progressive reduction towards the lesions [24]. These alterations were in keeping with our findings in the chronic EAE model (above), with persistent loss of Cx47 and Cx32 GJ even away from white matter lesions. Studies in highly active demyelination cases including acute MS lesions and neuromyelitis optica (NMO) showed that immunoreactivity for Cx43 was completely lost in highly degenerative GFAP positive astrocytes within the active lesion, while Cx43 was up-regulated in chronic lesions [110], reflecting the acute and chronic EAE findings (above).

The expression of oligodendrocytic Cx32 and Cx47 and their astrocytic partners Cx30 and Cx43 were also examined in cortical lesions and normal-appearing gray matter (NAGM) of MS patients [25]. Multiple sclerosis GM is characterized by more diffuse pathologic alterations than the classic WM lesions and a lack of infiltrating immune cells. We found widespread disruption of oligodendrocyte GJs and loss of O/A channels in the setting of chronic inflammation and astrogliosis favoring increased A/A connectivity. Our data obtained from the cortex showed overall marked alterations in glial GJs in MS NAGM and cortical lesions, which could have implications for the development and maintenance of tissue damage, synaptic transmission and cognitive deficits in MS patients [25].

Taken together, studies in chronic MS brain indicate widespread disruption of oligodendrocyte connexins both in lesions of the white and gray mater, as well as in NAWM and NAGM. These alterations are associated with diffuse inflammation and astrogliosis, whereby reactive astrocytes appear to be increasingly coupled with each other upregulating their own connexins, while at the same time they disconnect from oligodendrocytes. These chronic changes are likely to be preceded by loss of Cx43 in astrocytes during acute inflammatory events and secondary disruption of oligodendrocyte GJs, as shown in highly active MS and NMO cases ([110] and our own unpublished observations), in keeping with the time course demonstrated in experimental models.

Loss of Cx47 in oligodendrocytes and disconnection from astrocytes is likely to increase oligodendrocyte vulnerability, as directly demonstrated by the exacerbated EAE course and pathology in Cx32 KO and Cx47 KO mice (above) [23,27]. Furthermore, it may disrupt the differentiation of OPCs and their ability to re-myelinate in chronic MS brain. Additionally, the loss of Cx32 GJ along myelinated fibers in MS NAWM and perilesions may represent an important mechanism of secondary disease progression [111,112] since early axonal dysfunction has been demonstrated in still myelinated fibers of Cx32 KO mice in the peripheral nerves [46]. Overall, myelinated fibers with deficient formation of Cx32 GJ in MS NAWM may be more vulnerable to the inflammation [113]. Most importantly, based on our recent studies indicating a pro-inflammatory effect of GJ-deficient oligodendrocytes [27], loss of oligodendrocyte connexins in MS may not represent only a consequence of disease pathology, but may also drive inflammation and disease progression.

## The functions of connexin hemichannels and pannexin channels in oligodendrocytes

In addition to GJ channels, connexins form single membrane channels (hemichannels or connexons), that are not incorporated in GJs and are used to exchange small molecules, ions, and signaling molecules between the intra- and extra-cellular environments [114]. In detail, Cxs hemichannels, serve as autocrine/paracrine cellular communication pathways, allowing the transportation of ATP, NAD^+^, glutamate, and prostaglandins [115–117]. These channels can open in response to a variety of electrical and chemical signals. However, there is only limited information regarding the functional expression of hemichannels in oligodendrocytes [114]. A recent study revealed that Cxs hemichannels provide a major pathway for glucose entry into OPCs and oligodendrocytes [11]. Moreover, the glucose uptake via Cxs hemichannels was higher in OPCs (that do not express glucose transporter 1-GLUT1) and highly dependent on Ca^2+^. Interestingly, inhibition of these hemichannels resulted in a significant reduction of OPC proliferation suggesting that glucose uptake via Cx hemichannels is essential for the development of the oligodendrocyte lineage [11]. Based on these results it has been proposed that Cxs hemichannels in OPCs and mature oligodendrocytes are probably involved in both the maturation of oligodendrocytes but also in metabolic coupling and energy supply to neurons [11]. To our knowledge, there are no data available regarding the roles of Cx HCs in demyelinating diseases including MS.

Pannexins (Panxs) is another class of proteins expressed in the CNS that form channels (pannexons) [118], but not gap junctions, and are located at the cell surface [119]. Panxs include only three members, i.e., Panx1, Panx2, and Panx3. Even though pannexins do not share amino acid sequence homology with connexins, both protein families have similar secondary and tertiary structures [120]. Oligodendrocytes express Panx1 and the functional role of Panx1 channels in oligodendrocytes has been recently reported [121]. Panx1 channels are permeable to relatively large molecules, including ATP and other gliotransmitters [122]. Under pathological conditions, Panx1 channels become dysregulated leading to abnormal ATP transmission [122]. Panx1-mediated ATP release was reported in spinal cord oligodendrocytes in response to inflammatory mediators and to oxygen/glucose deficiency [121]. ATP is involved in different signaling platforms and induces excitotoxic cell death within the nervous system. In myelinated tissue, ATP initiates an excitotoxic cascade that promotes apoptosis of oligodendrocytes [121–123]. Oligodendrocyte death is one of the hallmarks of demyelinating diseases.

Recently, the role of Panx1 in the development and progress of EAE using WT and *Panx1* KO mice has been reported [124]. *Panx1* KO mice showed delayed onset of clinical signs of EAE and decreased mortality compared to WT mice, although *Panx1* KO and WT mice developed comparable EAE symptoms. In *Panx1* KO EAE mice, spinal cord inflammatory lesions were reduced during the acute phase of the disease. In addition, inhibition of Panx1 channels with mefloquine reduced the severity of both acute and chronic EAE. Thus, it has been proposed that a Panx1-dependent mechanism contributes to EAE progression and that inhibition of Panx1 delays and reduces clinical signs of EAE. The exact underlying molecular mechanisms remain to be elucidated. In another study, dysfunction and altered expression of the gene encoding Panx1 have been observed in WT EAE mice, while Panx1 mRNA expression levels were upregulated in both acute and chronic phases of EAE [125].

Overall, further studies are required to clarify the nature of the Cxs that participate in the activity of oligodendrocyte hemichannels and to elucidate the role of Cx- and Panx-based channels in the pathogenesis and development of demyelinating diseases.

## Conclusions

**Figure 3.**
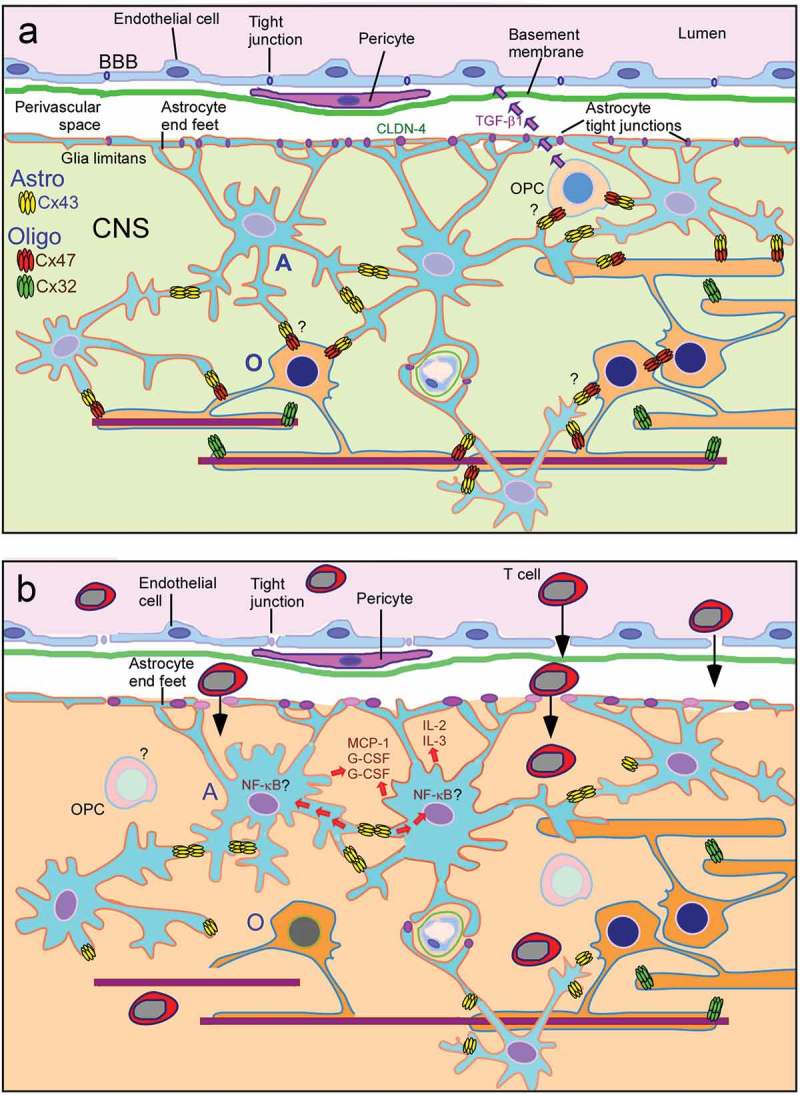
Diagram summarizing the role of glia connexin changes in demyelinating disorders. **a**. The top panel shows intracellular communication via connexin gap junctions between astrocytes and oligodendrocytes under normal conditions, and the possible effects of oligodendrocytes and their precursor cells (OPCs) on the blood-brain barrier (BBB) integrity, either directly through signaling or indirectly through the still to be clarified effects on astrocytes modifying their responses. **b**. The bottom panel demonstrates how the loss of oligodendrocyte and oligodendrocyte-astrocyte (O/A) GJs may result in the disruption of BBB and exacerbated neuroinflammatory response. Loss of the O/A Cx47-Cx43 channels is likely to lead on one hand to impaired homeostasis in oligodendrocytes and to accelerate demyelination and oligodendrocyte apoptosis under stress conditions. On the other hand, the disconnection of astrocytes from oligodendrocytes may alter the regulation of pro-inflammatory pathways within astrocytes, including the NFkB pathway, with further dysregulation of BBB and increased CNS inflammation and demyelination.

Insights from experimental models highlight the important roles of oligodendrocyte Cx32 and Cx47 in CNS demyelinating diseases and the integrity of the BBB/BSCB (Figure 3). As already demonstrated by human genetic disorders caused by dysfunction of oligodendrocyte connexins, and reproduced in relevant transgenic models, oligodendrocyte GJ connectivity is crucial for the development and maintenance of CNS myelin and axons. Furthermore, focusing on oligodendrocyte connexin pathology in post-mortem brain samples from MS patients revealed that the essential network of intra- and intercellular communication connecting myelin and oligodendrocytes to the glia syncytium is disrupted in chronic active and inactive MS lesions as well as in the NAWM, in cortical lesions and in NAGM [24–26,79,126]. Although it was initially thought that connexin pathology is more of a consequence of the inflammation and astrogliosis occurring in MS and EAE, subsequent findings from genetic models without induced inflammation, and further experiments with induced inflammatory demyelination in mice with connexin deficient oligodendrocytes, clearly demonstrated that loss of oligodendrocyte connexins can in turn drive a pro-inflammatory environment in the CNS, exacerbating EAE [27] and likely MS.

To conclude, recent studies from genetic and inflammation models, as well as from human disease, provide evidence for the crucial role of glial GJ channels in the regulation of homeostasis in the CNS including not only the development and maintenance of myelin, but also in BBB integrity and a timely and controlled response to inflammation and demyelination. Consequently, although the disruption of glial GJs may follow the inflammation and astrogliosis that occurs in MS and EAE pathology, it may also represent a causative factor, driving lesion formation, expansion, and finally disease progression [27]. Loss of connexins in oligodendrocytes as well as in OPCs may affect their potential for re-myelination, as they would have limited ability to connect to the glial network formed mainly by astrocytes. Further studies to better understand the role of oligodendrocyte connexins also in the remyelination phase would be useful and may provide insights into the relation between glial GJ connectivity and control of myelination and inflammation, offering novel therapeutic targets for MS.
